# Cognitive bias and how to improve sustainable decision making

**DOI:** 10.3389/fpsyg.2023.1129835

**Published:** 2023-02-28

**Authors:** Johan. E. (Hans) Korteling, Geerte L. Paradies, Josephine P. Sassen-van Meer

**Affiliations:** TNO Netherlands Organization for Applied Scientific Research, The Hague, Netherlands

**Keywords:** cognitive bias, nudging, decision making, behavioral influence, sustainability, sustainable behavior

## Abstract

The rapid advances of science and technology have provided a large part of the world with all conceivable needs and comfort. However, this welfare comes with serious threats to the planet and many of its inhabitants. An enormous amount of scientific evidence points at global warming, mass destruction of bio-diversity, scarce resources, health risks, and pollution all over the world. These facts are generally acknowledged nowadays, not only by scientists, but also by the majority of politicians and citizens. Nevertheless, this understanding has caused insufficient changes in our decision making and behavior to preserve our natural resources and to prevent upcoming (natural) disasters. In the present study, we try to explain how systematic tendencies or distortions in human judgment and decision-making, known as “cognitive biases,” contribute to this situation. A large body of literature shows how cognitive biases affect the outcome of our deliberations. In natural and primordial situations, they may lead to quick, practical, and satisfying decisions, but these decisions may be poor and risky in a broad range of modern, complex, and long-term challenges, like climate change or pandemic prevention. We first briefly present the social-psychological characteristics that are inherent to (or typical for) most sustainability issues. These are: experiential vagueness, long-term effects, complexity and uncertainty, threat of the status quo, threat of social status, personal vs. community interest, and group pressure. For each of these characteristics, we describe how this relates to cognitive biases, from a neuro-evolutionary point of view, and how these evolved biases may affect sustainable choices or behaviors of people. Finally, based on this knowledge, we describe influence techniques (interventions, nudges, incentives) to mitigate or capitalize on these biases in order to foster more sustainable choices and behaviors.

## 1. Introduction: The challenges of human welfare

Supported by science and technology, the world has undergone an explosively rapid change in only a few centuries which offers humanity enormous practical advantages in a large number of areas. Misery and misfortune as a result of food shortages, diseases, and conflicts that were previously considered unsolvable have been adequately tackled (Pinker, 2018). A large part of the world has achieved unprecedented economic growth, and on the waves of globalization, it is assumed that the less developed countries can in principle also benefit from this development (Harari, 2017). However, the technologies we use to increase our welfare today have effects, not only across the whole planet, but also stretching far into the future. In the wake of our pursuit of prosperity, humanity has created a number of new, and possibly even greater, problems. The economic growth, that has provided us with an abundance of food, energy, medicines, and living comfort, simultaneously destabilizes the ecological balance. To date, scientists have gathered broad and convincing evidence that under the influence of fossil energy consumption, there is a rapid global warming that may have devastating consequences for the health, wellbeing, and flourish of future generations. This includes sea level rise, droughts, floods, water shortage, and refugee flows (e.g., Meadows et al., 1972; Meadows, 1997; Kates and Parris, 2003; Millenium Ecosystem Assessment, 2005; Biermann et al., 2012; IPCC, 2013, 2014, 2021, 2022; Steffen et al., 2015). Other examples of ecological destabilization are: environmental pollution, pandemics, and massive extinction of plant and animal species. All these ecological imbalances pose a serious threat to the continued existence of the world and the survival of our civilization. In the Stone Age, the average person had around 4,000 cal. of energy per day at their disposal. Today, the average American uses around 230,000 cal., sixty times as much (Harari, 2017). To offer everyone in this world the same standard of living as persons living in the USA, we would need at least four planets, but we only have one (OECD, 2012). At the same time, the world seems hesitating to take decisive preventative action.^1^ So, despite that most scientists and an increasing number of politicians and citizens acknowledge these facts, this common understanding has not caused much change in our collective behavior. Humanity thus seems to lack the kind of rationality or wisdom that is needed to make substantial financial, social, or material changes in order to stop possible disasters that threaten long-term wellbeing, i.e., to create a world in which people can flourish and be happy.

### 1.1. Cognitive bias in sustainability issues

How can this be? Human decision making can be quite questionable at times. For example, it often seems to underestimate the long-term dangers of things like global warming and species extinction. This can make even major future threats seem insufficient motivation for determined action (Berger, 2009). In general, we see these types of typical, and often flawed, decision making patterns in many different contexts of our society (Eigenauer, 2018). For instance, Flyvbjerg (2009) showed that 9 out of 10 transportation infrastructure projects end up in large cost overrun, which did not improve over time, even over a period of 70 years. Other examples of persisting problems that for a major part follow from poor decision making are: improper and incorrect diagnoses as well as harmful patient decisions in medicine and health care (Croskerry, 2003; Groopman, 2007); overly optimistic growth assessments and ill-advised lending policies in global finance (Shiller, 2015); optimistic decision making in personal finance, like susceptibility to scams (Modic and Lea, 2013); against all knowledge continue a chosen course or investment with negative outcomes rather than alter it (Arkes and Blumer, 1985; Garland and Newport, 1991); perpetuating injustice through personal prejudice and unjust sentencing (Benforado, 2015); and accepting superstitions or conspiracy theories while rejecting scientific findings that contradict these beliefs (Yasynska, 2019).

In this article, we will focus on how the human brain and its evolved psychological characteristics affect people’s decision making. Effects of the workings of our brain and of our evolutionary heritage on decision making manifest most prominently in cognitive biases (Kahneman et al., 1982; Hastie and Dawes, 2001; Shafir and LeBoeuf, 2002; Haselton et al., 2005; van Vugt et al., 2014; Korteling et al., 2018). Cognitive biases can be generally described as systematic, universally occurring, tendencies, inclinations, or dispositions in human decision making that may make it vulnerable for inaccurate, suboptimal, or wrong outcomes (e.g., Tversky and Kahneman, 1974; Kahneman, 2011; Korteling and Toet, 2022). Well-known examples of biases are hindsight bias (once we know the outcome, we tend to think we knew that all along), tunnel vision (when we are under pressure, we tend to overfocus on our goal and ignore all other things that are happening), and confirmation bias (we tend to only see information that confirms our existing ideas and expectations). People typically tend to pursue self-interest at the expense of the community (Tragedy of the commons). We tend to over-value items we possess (Endowment effect) and we have a strong urge to persist in courses of action, with negative outcomes (Sunk-cost fallacy). What is more, biased decision making feels quite natural and self-evident, such that we are quite blind to our own biases (Pronin et al., 2002). This means we often do not recognize it, and therefore do not realize how our biases influence our decision making.

Cognitive biases are robust and universal psychological phenomena, extensively demonstrated, described, and analyzed in the scientific literature. In a wide range of different conditions, people show the same, typical tendencies in the way they pick up and process information to judge and decide. In line with their systematic and universal character, cognitive biases are also prominent in societal issues and policymaking (e.g., Levy, 2003; McDermott, 2004; Mercer, 2005; Baron, 2009; Flyvbjerg, 2009; Vis, 2011; Arceneaux, 2012; Shiller, 2015; Bellé et al., 2018). For example, Arceneaux (2012) has shown that in discussing political arguments, individuals are more likely to be persuaded by arguments that evoke loss aversion, even in the face of a strong counterargument. And it has been demonstrated in many instances that policy makers tend to make risk-aversive decisions when they expect gains, whereas when facing losses they accept taking more risk (e.g., McDermott, 2004; Vis, 2011).

There are already many publications on cognitive biases showing how human psychological tendencies underly the choices and behaviors of people (e.g., Kahneman et al., 1982; Shafir and LeBoeuf, 2002; Kahneman, 2011). There is also some literature on which biases and human mechanisms play a role in our difficulties with preventing climate change (e.g., Gifford, 2011; van Vugt et al., 2014; Marshall, 2015; Stoknes, 2015). However, there is still lack of insight into how biases play a role in the process of environmental policymaking and how this knowledge may be used to deal with the major systemic challenges that the modern world is confronted with. Despite their possible substantial effects on society and human wellbeing, cognitive biases have never been a serious matter of concern in the social and political domain (Eigenauer, 2018). In this paper, we will therefore analyze the constellation of psychological biases that may hinder behavioral and policy practices addressing sustainability challenges. We will also look for ways to mitigate the potential negative effects of biases through influence techniques, like nudging (e.g., Thaler and Sunstein, 2008).

### 1.2. The rationale and drawback of biases

Given the inherent constraints of our information processing system (i.e., the limited cognitive capacities of the human brain) our intuitive inclinations, or heuristics, may be considered effective, efficient, and pragmatic. And indeed, intuitive or heuristic decision making may typically be effective in; natural (primal) conditions with time-constraints, lack (or overload) of relevant information, when no optimal solution is evident, or when we have built up sufficient expertise and experience with the problem (Simon, 1955; Kahneman and Klein, 2009; Gigerenzer and Gaissmaier, 2011). In these cases, the outcomes of heuristic decision making may be quite acceptable given the invested time, effort, and resources (e.g., Gigerenzer et al., 1999).

The fact that heuristic thinking deals with information processing limitations and/or data limitations (Simon, 1955) does not alter the fact that many of our judgments and decisions may systematically deviate from what may be considered optimal, advisable, or utile given the available information and potential gain or risk (Shafir and LeBoeuf, 2002). This has been demonstrated by a large body of literature, showing how cognitive heuristics or biases may lead to poor decisions in a broad range of situations, even including those without complexity, uncertainty, or time constraints (Korteling et al., 2018). Imagine, for instance, a board of directors that has to decide about the continuation of a big project. Typically, the more they have invested so far, the less likely they are to pull the plug. This is not rational (and is therefore called the sunk cost fallacy), because what should matter is what the costs and benefits will be from this point forward, not what has already been spent. The Sunk-cost fallacy, like various other psychological biases affecting decision making, may continuously pop up in the world we live in. Examples are the Anchoring bias (Tversky and Kahneman, 1974; Furnham and Boo, 2011), Authority bias (Milgram, 1963), Availability bias (Tversky and Kahneman, 1973, 1974), and Conformity bias (Cialdini and Goldstein, 2004).

A large number of different biases have been identified so far and specific biases are also likely to occur in the domain of public decision making. By public decision making, we mean not only collective and democratic decision making, but also individual decision making. For different kinds and domains of decision making, different biases may occur. It may be expected that in decision making within the sustainability domain, certain (categories of) biases may more often occur than others. In this paper, we try to present the most relevant biases and the associated nudges, focusing on public decision making with regard to sustainability challenges.

## 2. Methods

Decision making in our modern society may be done on an individual basis, but may also involve many participants or stakeholders with their own perspectives and background, i.e., citizens, policy makers, company representatives, and interest groups (e.g., Steg and Vlek, 2009). To come to a comprehensive understanding of which psychological biases are likely to pop up in this context, we selected those biases that would likely be most prominent, given the typical (psychological) characteristics of sustainability issues. Next, we described interventions or influence techniques (incentives, nudges) to overcome, mitigate, or capitalize on these biases. This was done in three steps.

### Step 1: Defining psychological characteristics of sustainability problems

Sustainability issues have characteristics that may evoke certain biases. Here, we define “sustainability” as: a balanced development in which the exploitation of resources, the direction of investments, the orientation of technological development, and institutional change are all in harmony and enhance both current and future potential to meet long-term wellbeing. First, on the basis of the literature (e.g., Schultz, 2002; Steg and Vlek, 2009; van Vugt, 2009; van Vugt et al., 2014; Engler et al., 2018; Toomey, 2023) and a workshop with experts we defined a set of general social-psychologically relevant characteristics or factors, like “experiential vagueness” or “long-term effects” or “threat of the status quo” that are associated with most sustainability issues.

### Step 2: Biases per sustainability characteristic

Each characteristic of sustainability issues may relate to a few specific biases that may hamper sustainable choices and behaviors of people. For example, the long-term character of sustainability implies may be in conflict with our tendency to short-term thinking (Hyperbolic time discounting) or the tendency to underestimate both the likelihood of a disaster and its possible consequences, and to believe that things will always function the way they normally function (Normalcy bias). The subsequent identification of thinking tendencies and biases related to these characteristics was based on the literature entailing overviews of multiple biases (e.g., Korteling et al., 2020a), a Neuro-Evolutionary Bias Framework (Korteling et al., 2020a,b; Korteling and Toet, 2022), and on the literature on cognitive biases and sustainability challenges (e.g., Gardner and Stern, 2002; Penn, 2003; Fiske, 2004; Wilson, 2006; Steg and Vlek, 2009; van Vugt, 2009; van Vugt et al., 2014; Marshall, 2015; Engler et al., 2018).

### Step 3: Influence techniques per sustainability characteristic

Also, for each group of biases, some relevant intervention techniques that can be used, by for example government or policy makers, were briefly described. These interventions, incentives, or nudges, may be applied to mitigate the relevant biases or to capitalize on them for the purpose of stimulating decision making that is more in line with sustainability goals in the context of the current world. On the basis of a previous literature review (Korteling et al., 2021), we have chosen not to advocate specific educational approaches, aiming at bias mitigation training in order to foster sustainable decision making. Instead, our approach aims at interventions with regard to the context or environment in which people live order to promote more sustainable choices.

### Example of the approach

Finally, we will illustrate our approach with the help of an example: A conflict between personal versus community interest is a typical characteristic that is associated with sustainability issues. Natural selection has favored individuals who prioritize personal benefits over those of unrelated others (Hardin, 1968; van Vugt et al., 2014). This means that making choices in the public interest is often hindered by our personal interests (Step 1). Sustainability also often involves a trade-off between personal interests, such as driving a car or flying, against collective interests, such as fresh air and a peaceful environment. This conflict relates to the bias called the *Tragedy of the commons*, i.e., the tendency to prioritize one’s own interests over the common good of the community (Step 2). Because we share our genes with our relatives, this tendency may be countered by invoking kinship as a nudge. Pro-environmental actions or appeals may thus be more effective if they emphasize the interests of our ingroup, children, siblings, and grand-children (Step 3).

## 3. Most relevant psychological characteristics of sustainability challenges

Below, we list a set of prominent psychological characteristics that we consider relevant for sustainability issues. Although biases are inherent to the thinking and decision making in all people, it may be supposed that biases may differ depending on peoples’ places, functions, and roles in decision situations. On the other hand, there are many mutual influences and dependencies in the policymaking arena. Therefore, we have decided not to make clear distinctions between the specific roles people play in this arena. So, we do not discern biases for citizens, politicians or policy makers.

- *Experiential vagueness:* Sustainability problems are slowly and gradually evolving. Therefore, the impact of the issue is difficult, if not impossible, to perceive or experience directly with our body and senses. Our knowledge of the issue is largely built on indirect and abstract cognitive information, i.e., on conceptual reasoning, abstract figures, written papers, and quantitative models.
- *Long-term effects and future risk*: The negative consequences of green practices follow directly, whereas the positive aspects of green practices may emerge only after many years in the (far) future. The same counts for the positive consequences of not taking green action. In addition, sustainability concerns an unknown future with an abundance of possibilities that easily go beyond our imagination.
- *Complexity and uncertainty:* The sustainability issue is very complicated (socially, technically, logistically, economically) and even “wicked.” Being able to judge and reason over most topics within the field requires multi- and transdisciplinary knowledge. Sustainability challenges are (therefore) accompanied by a high degree of uncertainty about their future progression and how it should be tackled and addressed.
- *Threat to the status quo:* Many sustainability measures more or less have impact on (sometimes even threaten) our established way of living and basic societal infrastructure. When new measures have an impact on our “normal,” established way of living and basic societal infrastructure, this may be experienced as a threat that will result in losing our freedom and/or comfort (“fear of falling”).
- *Threat of social status*: Many environmental problems result from a desire to possess or consume as much as possible, instead of consuming “enough” for a good life. Consumptive behavior and high energy consumption are intrinsically related to high social status, which is something most people do not want to lose.
- *Social dilemma’s:* The sacrifices that have to be made in order to foster sustainability are mainly beneficial for the collective, whereas direct individual gains are often limited. In this “social dilemma,” humans tend to prioritize direct personal interests relative to more sustainable ones that benefit the planet.
- *Group pressure*: Norms, values, and standards for what is considered as ‘normal’ or what is considered “desirable” are determined and reinforced by group pressure. Also with regard to green choices, we are often more strongly influenced by the behaviors and opinions of our peers than by our personal views and attitudes toward conservation.

## 4. Biases and interventions per psychological sustainability characteristic

For each of the above-mentioned general psychological characteristics of sustainability issues, the next subsections will provide an analysis and inventory of the (kinds of) cognitive biases that are probably most relevant and critically involved in the associated public and political decision making processes. Finally, for each general characteristic, influence techniques (interventions) to mitigate or capitalize on the relevant/critical biases will be briefly described. These interventions are based on the literature concerning “psychological influence” (e.g., Jowett and O’Donnell, 1992; Cialdini, 2006; Adams et al., 2007; Cialdini, 2009; Hansen, 2013; Heuer, 2013; Korteling and Duistermaat, 2018; Toomey, 2023). The influence techniques have an informational nature. They can be utilized in public communication, education, and policy making, especially in communication to the public, in different forms of media. Because the biases mentioned show a great deal of overlap and similarity—it was more about groups or types of similar biases—we chose not to make explicit links between specific biases and the associated nudge.

### 4.1. Experiential vagueness

Social scientists have long been puzzled as to why people are so poor at recognizing environmental risks and ignore global environmental hazards (Slovic, 1987; Hardin, 1995). Such apathy is probably a product of our evolutionary heritage that produced a brain that is optimized to perform biological and perceptual-motor functions (Haselton and Nettle, 2006; Korteling et al., 2018; Korteling and Toet, 2022). For example, the vertebrate eye evolved some 500 billion years ago, compared to 50,000 years ago for human speech; while the first cave drawings are dated at 30,000 years, compared to the earliest writing system approximately 5,000 years ago (Parker, 2003; see also Grabe and Bucy, 2009). This comparatively more ancient visual perceptual and communicative apparatus enables us to quickly extract meaning from eye-catching images (Powel, 2017). In addition, there was always a tangible link between behavior and the environment. That is: if you do not eat, you will become hungry and search for food. If it starts raining, you may look for shelter in order to prevent becoming wet. A critical difference between the modern world and our ancestral environment is that we rarely see, feel, touch, hear, or smell how our behaviors gradually impact the environment (Uzzell, 2000; Gifford, 2011). Because our ancestors were not confronted with the relatively remote, slowly evolving, or abstract problems (Toomey, 2023), we probably are not well-evolved to be alarmed when confronted with potential or novel dangers that we cannot directly see, hear, or feel with our perceptual systems (van Vugt et al., 2014).

The human senses and nervous system show a gradual decrease in responsiveness to constant situations. In general, we are more sensitive to, and more easily triggered by, sudden changes and differences in the stimulus (contrasts). Because of this neural adaptation, we often may have difficulty with perceiving and appreciating slow and gradual processes of change. Therefore, the gradual changes that are implied in our environment, like global warming, are not very easily noticed. So, most people are generally not really alarmed by the gradual evolving and remote environmental challenges that the world is facing. This may contribute to the relatively low public interest in the issue of environmental threats such as global climate change, pollution of the oceans, extinction of species, the negative health effects of particulate matter, and decreasing biodiversity (Swim et al., 2011).

#### 4.1.1. Most relevant biases with regard to experiential vagueness

- *Experience effect*: the tendency to believe and remember things easier when they are experienced directly with our physical body and senses instead of abstract representations, like graphs and statistics, or text about scientific data (van Vugt et al., 2014).
- *Contrast effect*: having difficulty with perceiving and appreciating gradual changes or differences (instead of contrasting ones), such as gradually decreasing biodiversity and climate change (Plous, 1993).
- *Story bias*: the tendency to accept and remember more easily than simple or basic facts (Alexander and Brown, 2010).

#### 4.1.2. Interventions to mitigate these biases

##### Key: Make the consequences of possible ecological breakdown tangible

- To increase awareness of environmental threats people should experience by their senses (e.g., vision, sound, proprioception, and smell) how future situations will look and feel, e.g., by gaming, simulation or “experience tanks.” In raising and education, positive “nature experiences” can be used in order to promote a pro-environmental perspective of the world.
- People have difficulty with correctly perceiving and judging abstract figures. Quantitative data, tables, and numbers do not really make an impression and are thus easily ignored or forgotten.^2^ Make people therefore aware of environmental challenges using concrete examples and narratives that are related to real individuals with whom they can empathize and reinforce messages with vivid and appealing images, frames, and metaphors.
- Use pictures, animations, artist impressions, podcasts, and video’s instead of (or to support) written information.
- Focus on the concrete *consequences* of severe threats.
- Humans are evolved to love nature. So, increase the availability and number of opportunities (especially for city dwellers) to appreciate, experience and protect the healing value of the real nature, i.e., the fields, the woods, the waters, and the mountains (Schultz, 2002).
- Sustainability interventions that imply the loss of assets or privileges should proceed slowly, gradual, and in small steps. The more positive and rewarding aspects of transitions can be presented as more contrasting, sudden and discrete events.
- Narratives and stories consisting of coherent events and elements—real or imaginary—are more easily accepted and remembered than plain facts, which may be useful to create or enhance feelings of connectedness and commitment to pro-environmental initiatives.
- From a psycho-social perspective face-to-face communication is probably the richest (and most natural) form of communication and interaction. Use therefore face-to-face communication to promote pro-environmental behavior.

### 4.2. Long-term effects and future risk

Sustainable choices are often only rewarded in the long-term future, while the costs and sacrifices have to take place in the present. Given two similar rewards, humans show a preference for one that arrives sooner rather than later. So, humans (and other animals) are said to *discount* the value of the later reward and/or delayed feedback (Alexander and Brown, 2010). In addition, this effect increases with the length of the delay. According to van Vugt et al. (2014), our tendency to discount future outcomes may have had substantial benefits in primitive ancestral environments, suggesting it is an evolved psychological trait (Wilson and Daly, 2005). If our ancestors had put too much effort into meeting future needs rather than their immediate needs, they would have been less likely to survive and pass on their genes in the harsh and unpredictable natural environment in which they lived (Boehm, 2012). Human psychology is thus naturally formed to maximize outcomes in the here and now, rather than in the uncertain future (van Vugt et al., 2014). Thus people in modern societies still may weigh immediate outcomes much more heavily than distant ones (Green and Myerson, 2004). This preference for today’s desires over tomorrow’s needs—and the conflict between people’s desire for immediate rather than delayed rewards—may be the cause of the persistence of many environmental problems.

Our brain tends to build general conclusions and predictions on the basis of a (small) number of consistent, previous observations (inductive thinking). A typical and flawed inductive statement is: “Of course humanity will survive. Up to now, we have always survived our major threats and disasters.”^3^ Even in highly educated and experienced people, inductive reasoning may lead to poor intuitive predictions concerning the risks in the (long-term) future (Taleb, 2007). We tend to focus on risks that we clearly see, but whose consequences are often relatively small, while ignoring the less obvious, but perhaps more serious ones. Next to such poor statistical intuitions, we have a preference for optimistic perspectives. This leads us to ignore unwelcome information and to underestimate the severity and probability of future (environmental) challenges and hazards (Ornstein and Ehrlich, 1989). This may be especially devasting when considering rare and unpredictable outlier events with high impact (“black swans”). Examples of black swans from the past were the discovery of America (for the native population), World War I, the demise of the Titanic, the rise of the Internet, the personal computer, the dissolution of the Soviet Union, and the 9/11 attacks. Many people ignore possible rare events at the edges of a statistical distribution that may carry the greatest consequences. According to Taleb (2007), black swans (or “unknown-unknowns”) rarely factor into our planning, our economics, our politics, our business models, and in our lives. Although these black swans have never happened before and cannot be precisely predicted, they nevertheless need much more attention than we give them. Also global warming may trigger currently unknown climate *tipping points* when change in a part of the climate system becomes self-perpetuating beyond a warming threshold, which will lead to unstoppable earth system impact (IPCC, 2021, 2022).

#### 4.2.1. Most relevant biases related to long-term effects

- *Hyperbolic time discounting:* the tendency to prefer a smaller reward that arrives sooner over a larger reward that arrives later. We therefore have a preference for immediate remuneration or payment compared to later, which makes it hard to withhold the temptation of direct reward (Alexander and Brown, 2010).
- *Normalcy bias*: the tendency to underestimate both the likelihood of a disaster and its possible consequences, and to believe that things will always function the way they normally function (Drabek, 2012). By inductive reasoning, we fail to imagine or recognize possible rare events at the edges of a statistical distribution that often carry the greatest consequences, i.e., black swans (Taleb, 2007).
- *Optimism bias:* (Positive outcome bias, Wishful thinking): the tendency to overestimate the probability of positive (favorable, pleasing) outcomes and to underestimate the probability of negative events (O’Sullivan, 2015).

#### 4.2.2. Interventions to deal with these biases

##### Key: Bring the rewards of more sustainable choices to the present

- In general, immediate reinforcements are usually better recognized or appreciated and have more effect. Provide thus immediate rewards for green choices, e.g., through subsidy and tax policy, so that it pays more directly to make them.
- Bring long-term benefits in line with short-term ones. For example: investing in solar panels with a quick payback period, subsidizing the purchase of pro-environmental goods, or taxing the use of fossil fuels.
- Make people aware that we live in a world that inherently involves unpredictable and (system-) risks with high impact, e.g., like the corona pandemic. These risks may have severe negative consequences, maybe not yet for themselves in the short term, but much more for their beloved children and grandchildren.
- Present required changes as much as possible in terms of positive challenges, that is in terms of potential benefits rather than negative terms: a more “relaxed and natural way of life” instead of “costs of energy transition.” Green policy will deliver a stable and predictable future within the foreseeable future that makes prosperity and well-being possible.

### 4.3. Complexity and uncertainty

The modern global world we live in is very complex with many intricate causal relationships. Everything is connected to everything, making it very difficult to see what exactly is going on in this dense network and how the interplay of societal, technological, economic, environmental, and (geo)political forces develops. Our wealth and comfort are made possible by many “hidden” enablers, such as child labor in third world sweatshops and animal suffering out of sight in the bio industry. The complexity of interrelated and hidden causes, consequences, or remedies is also very prominent in sustainability issues. Sustainability issues are about by a fine-grained logistic infrastructure and sophisticated technological inventions and their massive application. For example, the energy transition involves complex socio-technical systems that usually involve a high degree of uncertainty about how this will ultimately work out. Our cognitive capacities to pick up and understand all this technical, statistical, and scientific information are inherently limited (e.g., Engler et al., 2018; Korteling et al., 2018). How can we intuitively calculate how much CO_2_ emission reduction is required and how much (or little) certain technical or economical interventions contribute to the reduction of greenhouse gases? Many people have also poor capacities for calculation and logic reasoning and a poor intuitive sense for coincidence, randomness, statistics, and probability reasoning (e.g., Monat et al., 1972; Sunstein, 2002; Engler et al., 2018). For instance, concepts like “exponential growth”—i.e., when the instantaneous rate of change of a quantity in time is proportional to the quantity itself—are generally poorly understood.

The inherent constraints of our cognitive system to collect and weight of all this information in a proper and balanced way may result in various biases preventing good judgment and decision making on the basis of the most relevant evidence. Our brain tends to selectively focus on specific pieces of information that ‘resonate’ with what we already know or expect and/or what associatively most easily pops up in the forming of judgments, ideas, and decisions (Tversky and Kahneman, 1974; Korteling et al., 2018; Toomey, 2023). The fact that other (possible relevant or disconfirming) information may exist beyond what comes up in our mind may be insufficiently recognized or ignored (Kahneman, 2011). This often may lead to a rather simplistic view of the world (e.g., populism). We trust and focus on what is clearly visible or (emotionally) charged, what we (accidentally) know, what we happened to see or hear, what we understand, what intuitively feels true, or what associatively comes to mind (the known-knowns). In contrast, we are rather insensitive to the fact that much information does not easily come to us, is not easily comprehensible, or simply is unknown to us. So we easily may ignore the fact that there usually is a lot that we do not know (The unknowns). This characteristic of neural information processing has been termed: *the Focus principle* (Korteling et al., 2018) or “What You See Is All There Is” (WYSIATI, Kahneman, 2011). An important consequence of this principle is that we tend to overestimate our knowledge with regard to complex issues about which we lack experience or expertise (Kruger and Dunning, 1999). A situation may also be deemed as too uncertain or complicated and a decision is never made due to the fear that a new approach may be wrong or even worse. An abundance of possible options may aggravate this situation rendering one unable to come to a conclusion. In sustainability challenges, people may thus be very motivated to improve the situation, but still can be hampered by uncertainty and lack of understanding to take action.

#### 4.3.1. Most relevant biases related to complexity and uncertainty

- *Confirmation bias*: the tendency to select, interpret, focus on and remember information in a way that confirms one’s preconceptions, views, and expectations (Nickerson, 1998).
- *Neglect of probability*: the tendency to completely disregard probability when making a decision under uncertainty (Sunstein, 2002).
- *Zero-risk bias*: The tendency to overvalue choice options that promise zero risk compared to options with non-zero risk (Viscusi et al., 1987; Baron et al., 1993).
- *Anchoring bias*: Biasing decisions toward previously acquired information. In this way, the early arrival of irrelevant information can seriously affect the outcome (Tversky and Kahneman, 1974; Furnham and Boo, 2011).
- *Availability bias*: the tendency to judge the frequency, importance, or likelihood of an event (or information) by the ease with which relevant instances just happen to pop up in our minds (Tversky and Kahneman, 1973; Tversky and Kahneman, 1974).
- *Focusing illusion*: the tendency to place too much emphasis on one or a limited number of aspects of an event or situation when estimating the utility of a future outcome (Kahneman et al., 2006).
- *Affect heuristic*: basing decisions on what intuitively or emotionally feels right (Kahneman, 2011).
- *Framing bias*: the tendency to base decisions on the way the information is presented (with positive or negative connotations), as opposed to just on the facts themselves (Tversky and Kahneman, 1981; Plous, 1993).
- *Knowledge illusion* (Dunning-Kruger Effect): the tendency in laymen to over-estimate their own competence (Kruger and Dunning, 1999).
- *Surrogation* (means-goal): the tendency to concentrate on an intervening process instead of on the final objective or result, e.g., concentrating on means *vs* goals or on measures *vs* intended objectives (Choi et al., 2012).
- Ambiguity effect: the tendency to avoid options or actions for which the probability of a favorable outcome is unknown (Baron, 1994).

#### 4.3.2. Interventions to deal with these biases

##### Key: Provide more information and education especially to better understand the environmental consequences of human decisions and actions

- Consistency is more convincing than quantity. We believe that our judgments are accurate, especially when available information is consistent and representative for a known situation. Therefore, conclusions based on a very small body of consistent information are more convincing for most people than much larger bodies of (less consistent) data (i.e., “The law of small numbers”).
- Repetition of a pro-environmental message has more impact than just *one* attempt. This exposure effect can be enhanced by using all possible communication channels and media.
- Start with providing information the positive way you want it to taken by the target audience. Later the message may be extended by the less favorable nuances and details.
- Provide better statistical education and training and improve the communication on uncertainty and risk. When it comes to numbers, quantities, and changes therein, focus on total amounts rather than on proportions.
- Make pro-environmental information (e.g., about actions, initiatives, techniques etc….) salient and conspicuous. Focus (in a simple visual way) on the severe *consequences* of global warming and biodiversity loss (desertification, crop failure, and famine, millions of homeless and displaced people, risk of wars) instead of on the complex underlying mechanisms and processes.
- Influence is unlikely to fail due to information that is not provided. Therefore, in setting up an information campaign, it is generally not needed to invest all efforts in providing maximum possible “evidence” that is intended to confirm the deception. Consistency is dominant. In general, clear, recognizable, and simple information will be most easily picked up and accepted.
- Influence and persuasion is not only determined by *what* is, or is not, communicated (i.e., the content) but also by *how* it is communicated or presented (i.e., the frame or form). These latter superficial aspects are more easily, intuitively, and quickly processed than the deeper content of the message. This “framing” can thus be very well exploited for influencing peoples’ choices. Each message can be framed in numerous ways. So it may be very effective to analyze how to wrap up a message in the way you want it to be taken.
- Different people value, and pick-up, different information at different levels. Therefore, communicate messages at different levels of understanding, from the direct immediate consequences for the individual (micro) to the overarching long-term consequences for the world of the future and for future generations (macro).
- Present and facilitate as much as possible “total solutions.” Which are tailor-made to the target audiences.

### 4.4. Threat of the status quo

A basic premise of evolution is that all organisms strive for the continuation of their existence. This not only concerns the existence *per se*, but also the maintenance of stable living conditions (that are instrumental to this ultimate goal). For this reason (under normal circumstances and to prevent unexpected risk), we tend to strive at maintaining the present situation and to remain consistent with previous patterns (default effect). So, we easily accept, or prefer, to continue on the path taken and to maintain the status quo (default options) and we are afraid of choosing alternative, options that may turn out suboptimal (Kahneman and Tversky, 1979; Johnson and Goldstein, 2003; Chorus, 2010). Energy transition, as a possible solution of a future problem, is by many people experienced as threatening, not only to our established comfortable way of living, but to our individual and social basic needs as well. A transition to more sustainable practices may thus cause bad feelings of losing security and possessions, sometimes termed “fear of falling.”

In line with this, people have an overall tendency to experience the disutility of giving up an object as greater than the utility associated with acquiring it (i.e., Loss aversion). Thaler (1980) recognized this pattern, and articulated it as such: people often demand much more to give up an object than they would be willing to pay to acquire it. This is called the Endowment effect. In contrast to what most authors on cognitive biases suppose, we here speculate that the emotions that we feel when we anticipate possible loss of our assets are not the *cause* of our bias to avoid loss. Instead, they are the *result* of our pervasive bias for self-preservation and for maintenance our (neurobiological) integrity (Korteling et al., 2018). So in brief: we often prefer to hold on to the current situation and to continue on previous (al) choices. As such, we default to the current situation or status quo.

#### 4.4.1. Most relevant biases related to threat of the status quo

- *Status Quo bias*: the tendency to maintain the current state of affairs (Samuelson and Zeckhauser, 1988).
- *Default effect*: the tendency to favor the option that would be obtained if the actor does nothing when given a choice between several options (Johnson and Goldstein, 2003).
- *Sunk cost fallacy* (also known as Irrational escalation or Concorde effect): the tendency to consistently continue a chosen course with negative outcomes rather than alter it. The effort previously invested is the main motive to continue (Arkes and Ayton, 1999).
- *System justification*: the tendency to believe that the current or prevailing systems are fair and just, justifying the existing inaccuracies or inequalities within them (social, political, legal, organizational, and economical) (Jost and Banaji, 1994; Jost et al., 2004).
- *Cognitive dissonance*: the tendency to search for and select consistent information in order to try to reduce discomfort when confronted with facts that contradict own choices, beliefs, and values (Festinger, 1957).
- *Fear of regret*: feeling extra regret for a wrong decision if it deviates from the default (Dobelli, 2011; Kahneman, 2011).
- *Loss aversion*: the tendency to prefer avoiding losses to acquiring equivalent gains. Loss takes an (emotionally) heavier toll than a profit of the same size does (Kahneman and Tversky, 1984).
- *Endowment effect*: the tendency to value or prefer objects that you already own over those that you do not (Thaler, 1980).

#### 4.4.2. Interventions to deal with these biases

##### Key: Make sustainable options the default or easiest choice and present them as a gains rather than losses

- Make desired pro-environmental choices and behavior the default (the normal standard) or easiest choice. For example, providing only reusable unless specifically request a single-use plastic shopping bag, or designing buildings and cities to make walking and biking more convenient.
- Encourage active participation can be a major tool for triggering cognitive consistency pressures to build more sustainable habits. In general: active participation signals commitment to subjects, increasing their likely identification with the message or goal of the persuasion. Subsequently, they will tend to make choices that are consistent with their previous—in this case pro-environmental—actions.
- Based on cognitive dissonance theory (Festinger, 1957), the expression of self-criticism in peer (discussion) groups is a major influence technique. Making people vocalize promises (or sins) in public drives subjects to remain consistent with their and words.
- We believe that our judgements are accurate, especially when available information is consistent and representative for a known situation. It is therefore always important to provide consistent information.
- People tend to focus on, interpret, and remember information in ways that *confirm* their existing ideas, expectations or preconceptions. Therefore, in order to create an open mind, it is better to start with undeniable, true evidence and take care to not to start with highly disputable information evidence. The more complicated and contradictory aspects can be tackled later.
- The first goal in any effort to change another person’s mind must be to ensure that the subject is at least seriously considering the desired alternative. This requires to start with strong and obvious evidence which fits into the target’s existing conceptions of the world. In contrast, starting with less dramatic evidence tends to be unsuccessful since the information will be ignored, unnoticed, forgotten, or misperceived.
- Present changes in terms of gains instead of losses and circumvent the loss felt by people when they are asked to invest funds and provide support to acquire the necessary funds for the transition.
- Create a story different from loss: what are we gaining? For example: more rest, less rat race. Do not address people as consumers, but as citizens, changemakers, parents, etc.

### 4.5. Threat of social status

People are more focused on relative status than absolute status. This is, for example, demonstrated by the fact that people find an increase in wealth relative to their peers more important than their absolute wealth (Diener and Suh, 2000). In an experimental setting, researchers found that when presented with financial options, most people chose to earn less in absolute terms, as long as they relatively earned more than their peers (Frank, 1985). Not unrelated to our status-seeking tendency, humans tend to consume more than they need. In many historical civilizations, we find a penchant toward (excessive) consumption and showing of materials and riches (Bird and Smith, 2005; Godoy et al., 2007). From an evolutionary point of view, such displays of status may be rooted in a social advantage (Penn, 2003; Saad, 2007; Miller, 2009). Ancestors who strived for improvement of their situation and who tried to do better than their peers, probably have passed their genes better than those who had a more comfortable attitude. The wry side effects, however, are that the tendency to seek status through material goods—nowadays more than ever—may contribute substantially to the production of waste and the depletion of nonrenewable resources. Because we seek relative wealth, as opposed to seeking an absolute point of satisfaction, we are not easily satisfied and we tend to persistently strive for ever more status and wealth. Whether it be our smartphone, our sense of fashion, or our household appliances, they all rapidly become outdated as soon as newer or more fashionable versions enter the horizon. As economists say: we compare ourselves continuously with our neighbors; we want to “keep up with the Joneses.” Finally, items that are scarce or hard to obtain have typically more perceived quality and status than those that are easy to acquire. So many environmental problems can therefore be the result of a conflict between status-enhancing overconsumption versus having enough for a good life. This ‘Hedonic treadmill’ is encouraged by commercials offering us a never ending stream of new products that should make us, in one way or the other, happy and thus hungry to buy more.

#### 4.5.1. Most relevant biases related to threat of social status

- Affective forecasting (Hedonic forecasting, Impact bias): the tendency to overestimate the duration and intensity of our future emotions and feelings regarding events, encouraging putting effort into favorable results (greed) and into avoiding threats (Wilson and Gilbert 2005).
- Hedonic adaptation (Hedonic treadmill): the tendency to quickly return to a relatively stable level of happiness despite major positive or negative life events (Brickman and Campbell, 1971).
- Social comparison bias: The tendency, when making decisions, to favor individuals who do not compete with one’s own particular strengths (Garcia et al., 2010).
- Scarcity bias: the tendency to attribute greater subjective value to items that are more difficult to acquire or in greater demand (Mittone and Savadori, 2009).

#### 4.5.2. Interventions to deal with these biases^4^

##### Key: Connect sustainable options and choices with concepts, persons or goods that emanate a high social status

- Frame pro-environmental choices or options (like solar panels, bikes, or electric cars) as status symbols that show good beliefs and an exemplary way of life.
- In contrast, frame counter-environmental options (mopeds, flying, and meat consumption) as unattractive or associate them with low-status.
- Use high-status and admired or popular influencers and celebrities to promote pro-environmental options, e.g., in social media campaigns.
- Educate people to assess their quality of life in absolute terms of health, freedom, and comfort instead of in relative terms towards ‘the Jonesses’.
- Present the benefits of environmental as scarce. This can be done, for example, by pointing out others (competitors) who want the same goods or by drawing attention to possible future supply problems.

### 4.6. Personal versus community interest

Individual self-interest is often in conflict with the interest of the whole group. This is generally conceptualized as a social dilemma. This dilemma is usually referred to as the *Tragedy of the Commons* story (Hardin, 1968). This hypothetical example demonstrates the effects of unregulated grazing (of cattle) on a common piece of land, also known as “the commons.” In modern economic terms, ‘commons’ are any shared or unregulated resources to which all individuals have equal and open access, like the atmosphere, roads, or even the fridge of the office. Searching for direct individual profit, most individuals increase their use or exploitation of these common resources, thereby unintentionally causing it to collapse (Hawkes, 1992; Dietz et al., 2003). According to Hardin (1968) and van Vugt et al. (2014) the human mind is shaped to prioritize their personal interests over collective interests because natural selection favors individuals who can gain a personal benefit at the expense of unrelated others. Of course, there are situations under which the collective benefit will be prioritized over that of the induvial. But the conditions under which the human mind is triggered to prioritize the collective good over its own are generally less prevalent (Hardin, 1968).

According to Dawkins (1976), natural selection is the replication of one’s genes, which often comes at the expense of the survival of others’ genes. Power is thereby often instrumentally used for self-interest at the cost of others. So, survival of the species is not what primarily matters. However, this prioritizing of self-interest is dependent on the relationship of the individual to the group. In tight-knit communities where the individual knows himself to be dependent on the community, his behavior will be in line with this dependency and more likely be in favor of the in-group’s interests. When the individual does not feel this connection to an in-group (community), he is probably more likely to prioritize self-interest. Evidence for this strategy is seen in social dilemma research showing that most individuals tend to make selfish choices when they interact with other people in one-shot encounters (Komorita and Parks, 1994; Fehr and Gächter, 2002; van Lange et al., 2013). The evolutionary tendency to let self-interest prevail at the expense of others has direct implications for environmental practice, which often concerns the overexploitation of limited resources, such as the oceans, natural areas, fish stocks, clean air, etc. Consequently, many sustainability problems result from this conflict between personal and collective interests.

#### 4.6.1. Most relevant biases related to personal versus community interest

- Tragedy of the commons (Selfishness and self-interest): the tendency to prioritize one’s own interests over the common good of the community (Hardin, 1968).
- Perverse incentive effect (Cobra effect): the tendency to respond to incentives in a way that best serves our own interests and that does not align with the beneficial goal or idea behind the incentives, which may lead to “perverse behaviors” (Siebert, 2001).
- Anthropocentrism: the tendency to take the own, human perspective as the starting point for interpreting and reasoning about all sorts of things, such as nature and other living animals (Coley and Tanner, 2012).

#### 4.6.2. Interventions to deal with these biases

##### Key: Introduce and present sustainable options as the most favorable and profitable

- Because we share our genes with our relatives, kinship may be a good motivator of pro-environmental behavior. Pro-environmental appeals may be more effective if they emphasize the interests of our ingroup, children, siblings, and grand-children.
- Create programs where pro-environmental choices result in direct personal (or business) gain, e.g., by proper incentives or rewards, like tax exemptions.
- Create close-knit, stable, and small communities to foster pro-collective behavior and cooperation.
- In all species, behaviors reinforced by rewards or positive feedback tend to be repeated (Thorndike, 1927, 1933), and the more reinforcement, the greater the effect. Therefore, multiple reinforcements on desired social choices increase the chance that this will remain the case or repeat itself in the future.

### 4.7. Group pressure

Social psychologists have long known that people tend to adapt to the choices and behavior of others (Asch, 1956). Our tendency of following the majority is adaptive since for most species, the costs of individual learning, through trial and error, are substantial (Simon, 1990; Richerson and Boyd, 2006; Sundie et al., 2006; Sloman and Fernbach, 2018). Also for our ancestors, living in uncertain environments it would probably be better to follow and copy others’ behavior than figuring things out for yourself (Kameda et al., 2003; Gorman and Gorman, 2016). This is therefore probably an ancient and natural adaptive tendency which may also help maintaining or strengthening a position within the social group (Korteling et al., 2020a). We thus easily follow leaders or people with high status and authority in groups. We adapt to people around us with which we feel connected, but have an aversion against strangers. We have difficulty being indebted to others and we like and support kind, attractive and agreeable people. This can lead, for example, to after-talk and blind copying of the behavior of others and the faithful following of persuasive and charismatic persons. In line with this, it has been found that green practices are more strongly influenced by the behaviors of our peers than by our personal attitudes toward conservation. For example, when people see that their neighbors are not conserving, they tend to increase their own energy consumption as well, even when they had been conserving energy in the past (Schultz et al., 2007). This herd behavior is unconscious, and is mediated by mirror neurons in the brain (Chartrand and Van Baaren, 2009). However, the unconscious nature of this herd behavior is often not acknowledged or even denied by the conformers themselves (Nolan et al., 2008) and is thus hard to battle. Our modern world is built on the basis of an enormous amount of unsustainable methods, tools, practices, and applications, so there is still a long way to go to achieve a sustainable world. Hence, the human tendency to copy the behavior of others and to regard other people’s behaviors as the norm and justification of undesirable behavioral choices can be very detrimental to the achievement of sustainable goals.

#### 4.7.1. Most relevant biases related to group pressure

- Bandwagon effect: the tendency to adopt beliefs and behaviors more easily when they have already been adopted by others (Colman, 2003).
- Conformity bias: the tendency to adjust one’s thinking and behavior to that of a group standard.
- Ingroup (−outgroup) bias: the tendency to favor one’s own group above that of others (Cialdini and Goldstein, 2004).
- Authority bias: the tendency to attribute greater accuracy to the opinion of authority figures (unrelated to its content) and to be more influenced by their opinions (Milgram, 1963).
- Liking bias: the tendency to help or support another person the more sympathetically they feel, which is largely determined by: kindness, attractiveness, and affinity (Cialdini, 2006).
- Reciprocity: the tendency to respond to a positive action with another positive action (“You help me then I help you”) and having difficulty being indebted to the other person (Fehr and Gächter, 2002).
- Social proof: the tendency to mirror or copy the actions and opinions of others, causing (groups of) people to converge too quickly upon a single distinct choice (Cialdini, 2006).

#### 4.7.2. Interventions to deal with these biases

##### Key: Use social norms and peer pressure to encourage sustainable choices and behaviors

- When a behavioral change is requested, it will probably be better to focus peoples’ attention on others who already show the desired pro-environmental behavior instead of educating people about the bad behavior of others.
- People can be seduced to choose for a certain option if they see this in many other people. So, present desirable pro-environmental behaviors as behaviors of the majority of the people (or at least large groups) people. Foster, for example, the desired behavioral choices by advertisements suggesting this behavior is already adopted by groups of people.
- Use people with authority, powerful people, and/or attractive people to promote pro-environmental behavior.
- Create feelings of commitment and indebtment for people who make sacrifices for the community in order to foster sustainability.

## 5. Discussion and conclusion

### 5.1. Biases and nudges

In the present paper we have described how ingrained cognitive biases in human thinking may counter the development of green policy practices aimed at fostering a more sustainable and livable world. We have focused our study on how the form, content and communication of information affects our decisions and behavior with regard to sustainability. The influence techniques advocated in this paper are informational and psychological interventions, incentives, and/or nudges that could be effective with regard to biased thinking in the context of the current modern world. In general, biased information processing has served us for almost our entire existence (e.g., Haselton et al., 2005; Korteling et al., 2018). However, these natural and intuitive thinking patterns may be very counterproductive for coping with the global and complex problems the world is facing today. The many possible incentives and nudges presented show that there are many ways to deliberately capitalize on biased thinking in people in order to promote more sustainable behavioral choices.

In previous publications we have explained how biases originate from ingrained neuro-evolutionary characteristics of our evolved brain (e.g., Korteling et al., 2018; Korteling and Toet, 2022). This neuro-evolutionary framework provides more fundamental explanations for human decision making than ‘explanations’ provided by most social- or psychological studies. These latter (social-) psychological explanations are more ‘proximate’ in terms of “limitations of information processing capacity” (Simon, 1955; Broadbent, 1958; Kahneman, 1973; Norman and Bobrow, 1975; Morewedge and Kahneman, 2010), two metaphorical “Systems of information processing” (Stanovich and West, 2000; Kahneman, 2003; Evans, 2008; Kahneman, 2011), “emotions” (Kahneman and Tversky, 1984; Damasio, 1994), “prospects” prospects (e.g., Kahneman and Tversky, 1979; Mercer, 2005). “lack of training and experience” (Simon, 1992; Klein, 1997, 1998). Our neuro-evolutionary bias framework explains in terms of structural (neural network) and functional (evolutionary) mechanisms the origin of cognitive biases, why they are so systematic, persistent, and pervasive, and why biased thinking feels so normal, natural, and self-evident. Given the inherent/structural (“neural”) and ingrained/functional (“evolutionary”) character of biases, it seems unlikely that simple education or training interventions would be effective to improve human decision making beyond the specific educational context (transfer) and/or for a prolonged period of time (retention). On the basis of a systematic review of the literature, this indeed appears the case (Korteling et al., 2021). When it comes to solving the problems of the modern world, it will probably be impossible to defeat or eliminate biases in human thinking. Thus, we should always be aware of the pervasive effects of cognitive biases and be modest about our cognitive abilities to solve complex long-term problems in an easy way.

So, the effects on decision making of bias-mitigation training interventions are likely to be rather ineffective, in the same way that it is difficult to get people to change their eating habits by persuading them that chocolate or meat does not taste good. What is more: denying the ultimate and deep-seated neuro-evolutionary causes of the particularities and limitations of human thinking, may hamper adequate development and usage of effective interventions. For example: if governments strive to decrease the demand for energy-inefficient jacuzzi baths, but they ignore the influence of human evolutionary biases, this might lead to an intervention strategy that fails. Perhaps the government would try to persuade people that buying energy-consuming baths is unwise for the future. But in the context of our tendency to discount the value of future consequences, such a strategy on its own is likely to be rather ineffective. It would probably be more effective to use our knowledge of cognitive biases to our advantage. For example, the fact that we compare ourselves to our peers (Social comparison) might lead to a campaign in which the purchase of sustainable solar panels or a sustainable heat pump or fancy e-bike is related to status and prestige. Likewise, it is better to convey pro-environmental messages in a simple, consistent, repetitive, and tangible way and to focus on the consequences (bad or good) of ones choices, rather than on complex intervening processes. Finally, it is better to communicate information about the many aspects of sustainability at different levels of understanding at the same time, i.e., from the instant aspects for the individual to the global consequences for the world of the future.

### 5.2. The ethics of nudging

Above we have listed tips and tricks to provoke “sustainable decision making.” But as we write this, we realize all the more that this knowledge of how biases work, can be used for all kinds of purposes. In the ‘wrong’ hands, this knowledge about biases can be used to manipulate or incite the population to destructive. That is not even speculative, history has already shown this over and over again. Fossil industries that succeeded in holding back measures against global warming, doctors recommending brands of cigarettes, smear campaigns that led to witch-hunts, and anti-Semitic propaganda during World War II are just a few examples.

There is a serious ethical issue with using our knowledge of biases to our advantage (e.g., Bovens, 2009; Raihani, 2013). Who decides whether it is ethical to nudge citizens and use our knowledge of evolutionary biases to steer the choices and behavior of people? It sometimes may seem obvious that it is a good thing if you want to prevent incitement to hatred and violence, genocide or destructive such as smoking. But there is also a gray area. In the current pandemic, for example, we see that governments are doing their best to silence dissenting voices “for a good cause.” But counter voices also represent the basis of a democratic constitutional state, where counter voices must always be welcomed. Can we afford to go beyond our democratic boundaries, by nudging our citizens, for the sake of the climate? Our thought on this is as follows: Democracy means that everyone is allowed to make their voice heard about the goals that you want to achieve as a society. This report is about how to make your voice heard more effectively. It provides tools that everyone (not just politicians and policy makers) can use, for better or for worse. This applies to any instrument, AI, weapons, robots, ICT, etc.… The evil is not in the instrument, but in the purpose for which it is used. If we democratically choose to achieve certain goals, then it can be deemed defendable that governments use those instruments as effectively as possible to achieve those goals. It leaves people still free to choose their own path and goals.

### 5.3. A vision-based agenda

Politics can ensure that we as humanity behave more sustainably. In that case, our societal and physical environment will have to be organized differently, for example with far-reaching legislation (eg CO2 tax), a different market-oriented economy and a different transport system. However, these changes are held back by our ingrained preferences for short-term thinking, maintaining the status quo, personal interest, or herd behavior, which may result in fears like losing jobs or losing freedom. These thinking tendencies and fears are exploited by the lobbies of many powerful (e.g. fossil) parties with vested interests. That is why we have to search for ways to get moving as a society. An important part of this is managing well-being, and thereby discovering that there are ways to live sustainably, and also to be happy. This means that, more than ever, there is a need for knowledge and a substantiated vision about the core values that represent us, as humans, and our world, about who we are, how we want to live and where we want to go. This is not just a vision with long-term goals for human well-being, but also one that builds on our natural needs and that takes into account the hidden and inherent systemic risks of the modern, globalized world. This is essential in determining the course and the agenda for the future of humanity.

## Data availability statement

The original contributions presented in the study are included in the article/supplementary material, further inquiries can be directed to the corresponding author/s.

## Author contributions

The literature search, analysis, conceptual work, and the writing of the manuscript was done by JEK. GP provided knowledge and information concerning sustainability. JM critically reviewed the manuscript several times. All authors contributed to the article and approved the submitted version.

## Conflict of interest

The authors declare that the research was conducted in the absence of any commercial or financial relationships that could be construed as a potential conflict of interest.

## Publisher’s note

All claims expressed in this article are solely those of the authors and do not necessarily represent those of their affiliated organizations, or those of the publisher, the editors and the reviewers. Any product that may be evaluated in this article, or claim that may be made by its manufacturer, is not guaranteed or endorsed by the publisher.

## Footnotes

^1^The problem of climate change was put on the agenda by the Club of Rome, with their report *Limits to Growth* (Meadows et al., 1972). Since then numerous countries have agreed that action is needed. Climate goals were set numerous times, of which the last two were the Paris climate goals (Paris Climate Conference, COP21, 2015, and COP26 in Glasgow, 2021).

^2^Although exact “numbers” may sometimes provide information with an aura of objectivity and certainty.

^3^However, most human-like races, such as the Neanderthals, are now extinct and real major threats of humanity are those of a globalized world (which only exists for less than a couple of centuries) such as nuclear or biochemical weapons, global warming, or pandemics.

^4^Governments will want to consider the ethical preconditions and repercussions of these forms of nudging before engaging in it. Though it is a widely applied strategy in our neoliberal system where commercial advertisements are deemed acceptable to nudge the potential customer into buying their product. However, governments should uphold important ethical guidelines that concur with our values of freedom of choice and democracy. For a more in depth study of this, please read, e.g., van Vugt (2009) and Raihani (2013).
